# Dermoid and Ovarian Cysts

**Published:** 1878-10

**Authors:** C. H. Eames

**Affiliations:** East Saginaw


*ART. III.—Dermoid and Ovarian Cysts— Uterine Fibroid Tumor.* Cases occurring in the practice of Dr. B. B. Ross, East Saginaw, Mich. Reported by Dr. C. H. Eames.

Mrs. C., aged 40, Canadian; has always been a delicate woman; is the mother of five children, the last of which was born in June, 1878, and was a puny child, living but a few weeks. She first noticed a tumor in right iliac fossa six years ago, which was then as large as a small orange, and increased, but slowly, until date of last pregnancy, at which time it was pushed up into right hypochondrium, and began to grow rapidly, menstruation extremely painful and profuse since then, confining her to the bed. For several months she has been unable to evacuate the bowels or allow the escape of flatus, except by getting down on her hands and knees. Ovarian tumor was diagnosed, and on July 30th, in the presence of a number of physicians, Dr. Ross removed an unilocular atheroma—cyst of right ovary, weighing 15 pounds, and a smaller multilocular cyst of left ovary. The atheroma cyst contained a number of well formed teeth and a mass of hair. The multilocular tumor consisted of six small cysts, containing a serous straw-colored fluid, and one with atheroma-pulp.

The tumors were removed by enucleation, as performed by Prof. Miner, of Buffalo, the peritoneal capsule being dropped back into the abdomen after the bleeding (which was very slight) had ceased, no vessel demanding ligation appeared, and but two small points requiring torsion.

The abdominal wound was closed with silk sutures, which also included the peritoneum, and united throughout by first intention. The temperature at no time rose above 102·4° F., which occurred on the third day after the operation, at 2 A. M., pulse then 120. The patient sat up on the twelfth day, and was discharged on the thirtieth, in excellent spirits. It may be well to mention the existence of a loud presystolic heart murmur, with soft and irregular pulse in this case and that chloroform (Squibbs) was used for anæsthesia, producing no untoward symptoms.

Mrs. H., Canadian, aged 45, has borne three children, the last 12 years ago. On January 20th, 1877, Dr. Ross was called in consultation with Dr. J. R. Jones, of Midland. Patient anæmic and much emaciated; pulse 98, soft and irregular; lower extremities œdematous; heart not perceptibly enlarged, soft systolic murmur and rather feeble first sound; no history of rheumatism, urine albuminous at all times. Profuse leucorrhœa. Her health had been good until six months previously, when she noticed a tumor well down in the vagina, which she called "falling of the womb," and commenced to suffer from profuse loss of blood at menstrual periods.

Examination revealed a fleshy tumor, hard as a child's head, and completely filling the vagina, protruding slightly therefrom. Artificial aid is required for evacuation of bowels and bladder. Diagnosis, fibro-polyp of uterus. On January 22d the patient was anæsthetised with chloroform and an attempt made to pass a wire ecraseur around the pedicle, which was unsuccessful, for lack of room, the capsule of the tumor was thereupon cut open, and the mass cut away with curved scissors "piece-meal." When the tumor had been reduced in size as much as possible by this means, a blunt hook was introduced over the finger, within the capsule, and passed around the pedicle, and by firm traction the remaining portion was torn loose. The sack was then removed with the scissors, and found to have sprung from the posterior wall of the convix, immediately within the os. The patient rallied well from the chloroform, and on the sixth day sat up in bed for her meals When sitting up at breakfast on the tenth day, and without giving any warning, she fell back and expired. Any opinion concerning the cause of death in this case must be mere conjecture; but Dr. Ross is inclined to attribute the unfortunate result to an over-distended fatty heart, or perhaps to embolism.

Mrs. L., American, aged 34, has been married five years and never pregnant. She had always menstruated normally, until January, 1877, when dysmenorrhœa and metrorrhagia commenced, and have since become very much aggravated, especially the latter, confining her to the bed for nearly half the month. In the spring of 1877 she noticed a swelling in the hypogastrium; she was treated by a Homœopath until June of the same year, when Dr. Ross was called. The patient was then much exsanguinated and very nervous. An examination revealed an intramural fibroid tumor, occupying the anterior wall of the uterus and extending two-thirds of the way from the pubic crest to the umbilicus, also involving the vaginal portion of the cervix for three-fourths of its circumference. The sound passed to the depth of six inches, and showed the uterus to be slightly anteverted. The persevering use of sponge tents failed to dilate the cervical canal sufficiently to admit the finger, and four Molesworth's dilators were ruptured in the attempt with little better success. The patient was placed under the influence of chloroform and a long probe pointed knife passed into the uterine cavity, using the finger as a guide; two incisions were then made to the depth of an inch into the body of the tumor, extending from the fundus uteri to the internal os, and meeting in a V shape. The tumor creaked under the knife like cartilage; slight hemorrhage ensued and was easily checked.

Nothing of note occurred until the third day, when the patient became slightly feverish and complained of a sensation of heat in the pelvis. In a day or two a sero-purulent discharge appeared, and soon shreds of fibrous tissue and "pieces," some of which were as large as a walnut, commenced to come away through the now easily dilated os. Injections of chlorinated soda and warm water were ordered; a copious discharge continued for two weeks, and at the end of a month the patient was discharged, the uterine tumor having disappeared, and she rapidly regaining strength and health.

East Saginaw, Sept. 10th, 1878.

